# Supplementary material for: Comparison of visual performance between bifocal and extended-depth-of-focus intraocular lenses
Source: PLoS One. 2023 Jul 13;18(7):e0288602. doi: 10.1371/journal.pone.0288602 (PMC10343039; doi:10.1371/journal.pone.0288602)
Supplement: S1 Table — Patient demographics and pre/postoperative visual parameters (A, B). For categorical data, each category and its count and frequency are shown, and the two-sided Fisher’s exact test was used to compare categorical data across the bifocal and EDOF IOL groups. For numerical data, the means and standard deviations are shown, and the two-sided Mann–Whitney test was used to compare numerical data across the bifocal and EDOF IOL groups. Parameters in the bifocal and EDOF groups used to adjust the linear regression model are shown in (B). Age, sex, axial length (at the time of surgery), subjective refraction spherical equivalent (SE), subjective refraction cylinder (CYL), corneal astigmatism (keratometric cylinder), corneal higher-order aberrations (astigmatism, total higher-order aberration (HOA), third-, fourth-, trefoil, coma, tetrafoil, second-order astigmatism (2nd Astig), and spherical, scaled to a pupil size of 4 mm/6 mm). CYL, subjective refraction cylinder; HOA, higher-order aberration; WF_4_post_C, wavefront_4_post_corneal; SE, subjective refraction spherical equivalent. (ZIP) [file pone.0288602.s001.zip › S1B_Table.docx]

| (A) Categorical variable | | | | | |
| --- | --- | --- | --- | --- | --- |
|  |  | N (%) |  |  | |
| Variable | Levels | ZMB00 | ZXR00V | p value (Wald test) | |
| Sex | F/M | 518 (78.1)/145 (21.9) | 39 (37.1)/66 (62.9) | 6.449E-18** | |
| (B) Continuous variables | | | | | |
|  | | N, Mean ± SD |  | |  |
| Variable | | ZMB00 | ZXR00V | | p value (Wald test) |
| Age | | 663, 67.043 ± 7.809 | 105, 67.810 ± 6.891 | | 4.344E-01 |
| SE | | 1069, 0.229 ± 0.448 | 185, -0.241 ± 0.524 | | 3.012E-28** |
| CYL | | 849, -0.785 ± 0.397 | 185, -0.574 ± 0.504 | | 1.291E-10** |
| Corneal astigmatism | | 525, -0.731 ± 0.413 | 207, -0.674 ± 0.398 | | 5.831E-02 |
| Axial length | | 1326, 24.045 ± 1.574 | 210, 24.379 ± 1.622 | | 1.002E-03** |
| WF_4_post_C | |  |  | |  |
| Astigmatism | | 953, -0.873 ± 0.501 | 180, -0.863 ± 0.540 | | 5.333E-01 |
| Total HOA | | 953, 0.204 ± 0.105 | 180, 0.201 ± 0.096 | | 4.377E-01 |
| Third | | 953, 0.174 ± 0.100 | 180, 0.170 ± 0.092 | | 5.342E-01 |
| Fourth | | 953, 0.098 ± 0.054 | 180, 0.096 ± 0.054 | | 1.339E-01 |
| Trefoil | | 953, 0.131 ± 0.086 | 180, 0.126 ± 0.076 | | 5.269E-01 |
| Coma | | 953, 0.100 ± 0.075 | 180, 0.102 ± 0.074 | | 8.648E-01 |
| Tetrafoil | | 953, 0.059 ± 0.045 | 180, 0.062 ± 0.046 | | 5.519E-01 |
| 2^nd^ Astig | | 953, 0.039 ± 0.030 | 180, 0.044 ± 0.037 | | 2.147E-01 |
| Spherical | | 953, 0.048 ± 0.047 | 180, 0.037 ± 0.039 | | 5.681E-04** |
| WF_6_post_C | |  |  | |  |
| Astigmatism | | 868, -0.623 ± 0.433 | 143, -0.624 ± 0.512 | | 9.737E-01 |
| Total HOA | | 868, 0.580 ± 0.435 | 143, 0.591 ± 0.815 | | 3.236E-02* |
| Third | | 868, 0.390 ± 0.310 | 143, 0.395 ± 0.500 | | 4.873E-01 |
| Fourth | | 868, 0.368 ± 0.249 | 143, 0.369 ± 0.461 | | 8.942E-04** |
| Trefoil | | 868, 0.278 ± 0.236 | 143, 0.273 ± 0.363 | | 3.206E-01 |
| Coma | | 868, 0.245 ± 0.235 | 143, 0.263 ± 0.361 | | 6.043E-01 |
| Tetrafoil | | 868, 0.169 ± 0.180 | 143, 0.177 ± 0.302 | | 2.964E-01 |
| 2^nd^ Astig | | 868, 0.100 ± 0.158 | 143, 0.122 ± 0.292 | | 3.072E-01 |
| Spherical | | 868, 0.274 ± 0.162 | 143, 0.268 ± 0.232 | | 1.626E-03** |
| Pupil diameter, post | | 980, 4.480 ± 0.871 | 182, 4.156 ± 0.814 | | 2.080E-05** |

**Table S1B.** Parameters in the bifocal and EDOF groups used to adjust the linear regression model: age, sex, axial length (at the time of surgery), subjective refraction spherical equivalent (SE), subjective refraction cylinder (CYL), corneal astigmatism (keratometric cylinder), corneal higher-order aberrations (astigmatism, total higher-order aberration (HOA), third-, fourth-, trefoil, coma, tetrafoil, second-order astigmatism (2^nd^ Astig), and spherical, scaled to a pupil size of 4 mm/6 mm). For categorical data, each category and its count and frequency are shown, and Fisher’s exact tests were used to compare categorical data for the bifocal and trifocal IOLs. For numerical data, the mean and standard deviation are shown, and the Mann-Whitney test was used to compare numerical data for the bifocal and trifocal IOLs. * p<0.05, **p<0.002 (=0.05/25) SE: subjective refraction spherical equivalent; CYL: subjective refraction cylinder; WF_4_post_C: wavefront_4_post_corneal; HOA: higher-order aberration.
