# Supplementary figures and images for: Comparison of visual performance between bifocal and extended-depth-of-focus intraocular lenses
Source: PLoS One. 2023 Jul 13;18(7):e0288602. doi: 10.1371/journal.pone.0288602 (PMC10343039; doi:10.1371/journal.pone.0288602)

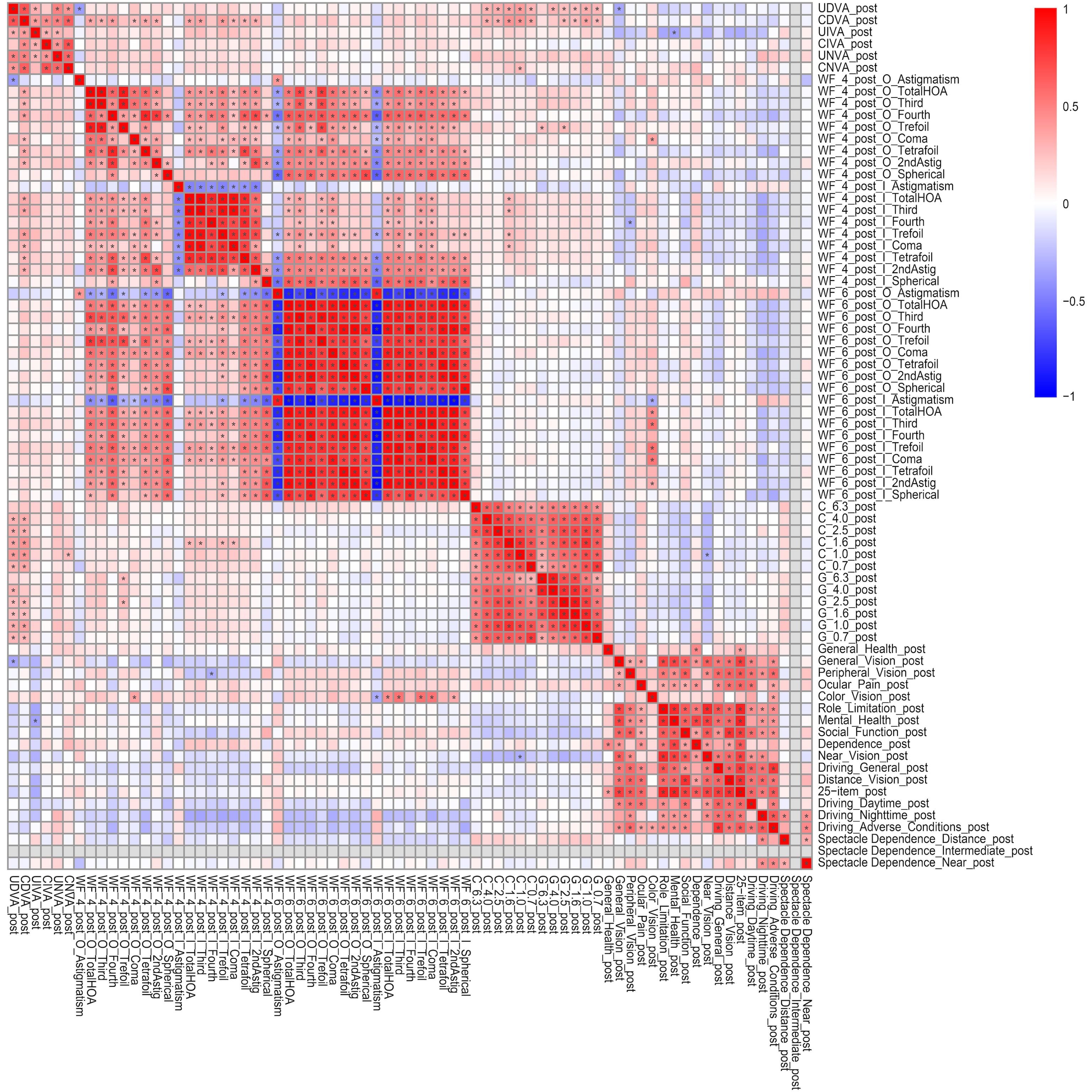

Supplement: S1 Fig — Pearson’s correlation coefficients were adjusted by multiple regression with the explanatory variables in S1B Table. The asterisk * in this figure indicates a significant correlation between two parameters at p<0.00002 after a Bonferroni correction. A two-sided t test was conducted to evaluate the significance of differences between the two groups. The sample size for each parameter is shown in S3C Table. The illustration was created using a commercially available software program (https://cran.r-project.org/web/packages/pheatmap/pheatmap.pdf) [23]. (TIF) [file pone.0288602.s005.tif]

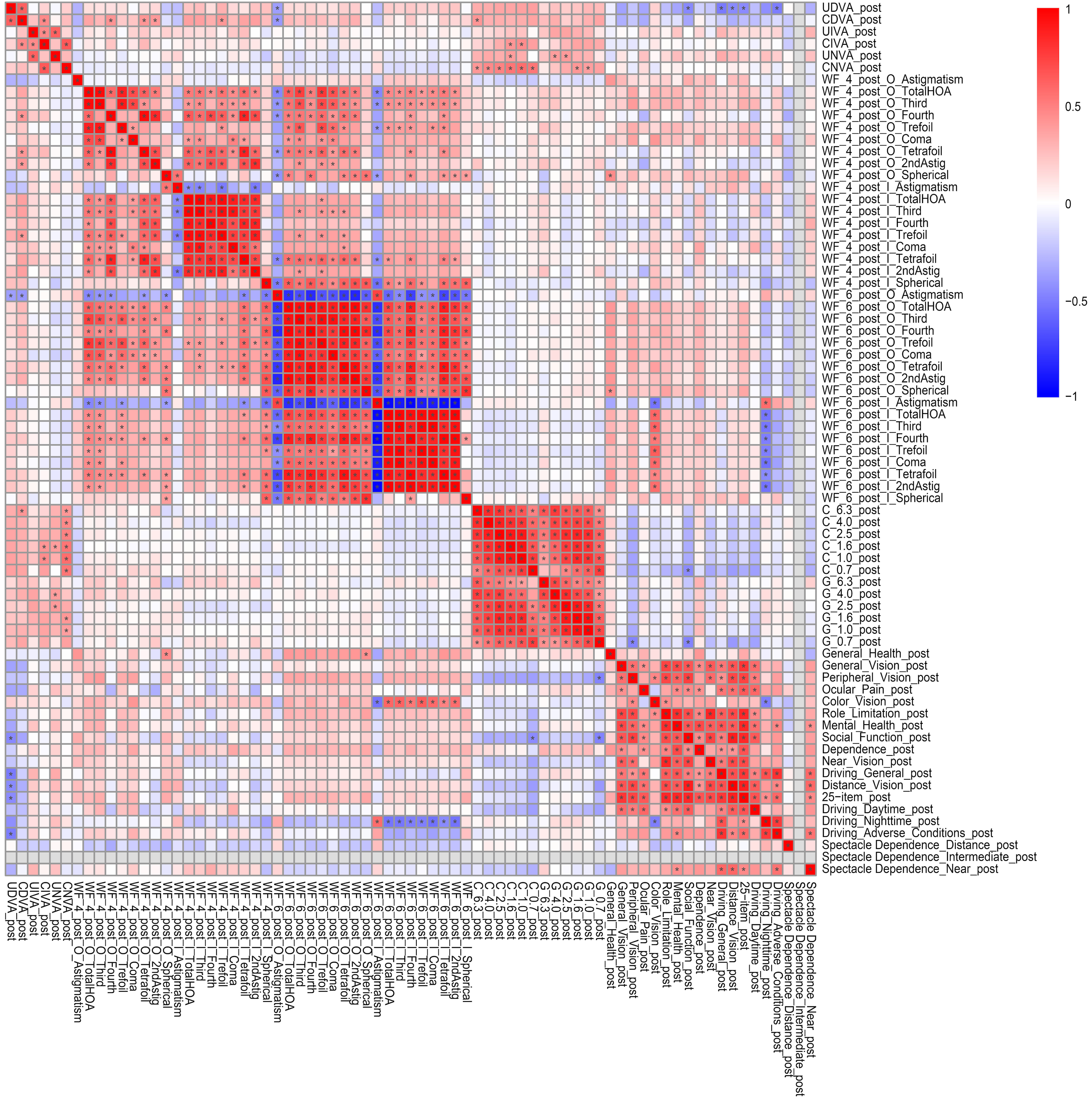

Supplement: S2 Fig — Pearson’s correlation coefficients were adjusted by multiple regression with the explanatory variables in S1B Table. The asterisk * in this figure indicates a significant correlation between two parameters at p<0.00002 after a Bonferroni correction. The two-sided t test was applied to evaluate the significance of differences between the two groups. The sample size for each parameter is shown in S3C Table. The illustration was created using a commercially available software program (https://cran.r-project.org/web/packages/pheatmap/pheatmap.pdf) [23]. (TIF) [file pone.0288602.s006.tif]
